# Multi-Modal Feature Selection with Feature Correlation and Feature Structure Fusion for MCI and AD Classification

**DOI:** 10.3390/brainsci12010080

**Published:** 2022-01-05

**Authors:** Zhuqing Jiao, Siwei Chen, Haifeng Shi, Jia Xu

**Affiliations:** 1 School of Computer Science and Artificial Intelligence, Changzhou University, Changzhou 213164, China; jzq@cczu.edu.cn (Z.J.); chensiwei1223@163.com (S.C.); 2 Department of Radiology, Changzhou Second People’s Hospital, Nanjing Medical University, Changzhou 213003, China; 3 School of Microelectronics and Control Engineering, Changzhou University, Changzhou 213164, China; 4 School of Medicine, Ningbo University, Ningbo 315211, China

**Keywords:** feature correlation, feature structure fusion, multi-modal, classification, feature selection

## Abstract

Feature selection for multiple types of data has been widely applied in mild cognitive impairment (MCI) and Alzheimer’s disease (AD) classification research. Combining multi-modal data for classification can better realize the complementarity of valuable information. In order to improve the classification performance of feature selection on multi-modal data, we propose a multi-modal feature selection algorithm using feature correlation and feature structure fusion (FC2FS). First, we construct feature correlation regularization by fusing a similarity matrix between multi-modal feature nodes. Then, based on manifold learning, we employ feature matrix fusion to construct feature structure regularization, and learn the local geometric structure of the feature nodes. Finally, the two regularizations are embedded in a multi-task learning model that introduces low-rank constraint, the multi-modal features are selected, and the final features are linearly fused and input into a support vector machine (SVM) for classification. Different controlled experiments were set to verify the validity of the proposed method, which was applied to MCI and AD classification. The accuracy of normal controls versus Alzheimer’s disease, normal controls versus late mild cognitive impairment, normal controls versus early mild cognitive impairment, and early mild cognitive impairment versus late mild cognitive impairment achieve 91.85 ± 1.42%, 85.33 ± 2.22%, 78.29 ± 2.20%, and 77.67 ± 1.65%, respectively. This method makes up for the shortcomings of the traditional multi-modal feature selection based on subjects and fully considers the relationship between feature nodes and the local geometric structure of feature space. Our study not only enhances the interpretation of feature selection but also improves the classification performance, which has certain reference values for the identification of MCI and AD.

## 1. Introduction

Alzheimer’s Disease (AD) is a neurological disorder associated with memory and mobility impairment and resulting in loss of cognitive function. With the aging of society, more and more elderly people are facing this disease. Studies have shown that the prevalence of AD in developing countries is much higher than that in developed countries [1]. Early mild cognitive impairment (EMCI) and late mild cognitive impairment (LMCI) is an intermediate state between healthy normal people and Alzheimer’s patients, and MCI gradually develops into AD with the development of the disease. Thus, determining how to accurately classify MCI and AD is of great significance.

In daily diagnosis, we can obtain massive amounts of medical image data with different structures and types. It helps us to observe the same subject from different perspectives and strengthen the understanding of the disease pathogenic factors. Traditional single-modal data only start from medical image data and observe the subjects from a single perspective. Obviously, the information complementarity between different modal data is ignored. This is bound to result in the acquired features not being comprehensive enough, affecting the final classification results. By observing the subjects with multi-modal data, we can understand the pathogenic factors of the disease more comprehensively. For example, Zhang et al. [2] combined Magnetic Resonance Imaging (MRI), Positron Emission Tomography (PET), and Cerebrospinal Fluid (CSF) data for feature selection. Li et al. [3] adopted two imaging techniques, Arterial Spin Labeling (ASL) and Blood Oxygen Level-Dependent Functional Magnetic Resonance Imaging (BOLD-FMRI), to conduct MCI classification and select features with good characterization. The results show that the classification effect of these two studies is better than that of single-modal data. Structural Magnetic Resonance Imaging (sMRI) and PET have been widely adopted in multi-modal feature selection [4,5,6,7]. These two modes can simultaneously obtain the structural and functional features of the brain, which can enhance the ability of feature description and facilitate feature expression.

For MCI and AD classification, the most important point is to carry out joint feature selection for the features extracted from multiple modal data. It is essential to screen out the features associated with the disease and improve the classification performance while reducing the feature dimension. In machine learning, feature selection algorithms can be roughly divided into filtering [8], wrapping [9], and embedded [10,11]. Embedded feature selection, which is widely applied, combines the learner with the feature selection process, and automatically completes feature selection when the learner learns. Regularization techniques are often applied to embedded feature selection algorithms. For example, the Lasso algorithm [12] uses $L_{1}$-norm regularizer to achieve feature selection effect with sparse feature weight vectors. Among the existing embedded feature selection algorithms, multi-task learning is often used for feature selection related to disease [13,14,15,16]. Its advantages are that it can reveal the potential common characteristics between different tasks, carry out information sharing between tasks, and has good generalization. For example, Jie et al. [17] obtained manifold structures of different modal data by combining manifold learning and multi-task learning, effectively combining information complementarity among multi-modal data; Lei et al. [15] adopted a new regularization to reduce rank relaxation based on multi-task learning, which can better carry out feature selection and reduce redundant features. In recent studies, Shao et al. [18] introduced hypergraph learning derived from multi-task learning and proposed a feature selection algorithm based on hypergraph to reflect the high-order relationship between subjects through the hypergraph Laplacian matrix.

However, the above methods only consider the potential relationship between subjects in different modalities or in the same modality, and do not satisfactorily consider the internal relationship between different modalities or different features in the same modality. Therefore, we propose a multi-modal feature selection with feature correlation and feature structure fusion that applies to MCI and AD classification. First, features are extracted from sMRI and PET data, and the correlation coefficient matrix between different modal features is converted into feature correlation regularization by weighted sum; Then, based on manifold learning, the feature matrix is fused, and feature structure regularization is constructed. A low-rank constraint is added based on the multi-task learning model, and two regularizations are embedded into the improved model to obtain the final feature selection model. The multi-modal features are selected by the proposed model, and the selected features are linearly fused into a support vector machine (SVM) for classification, and the final classification results are obtained. Then, the effects of different feature correlation calculation methods, the fusion coefficients of the feature matrix, and different regularized weight coefficients on classification performance are discussed. Finally, the brain regions corresponding to the selected features are analyzed to find the discriminative brain regions affected by MCI and AD diseases respectively.

## 2. Materials and Methods

### 2.1. Research Framework

**Figure 1:**
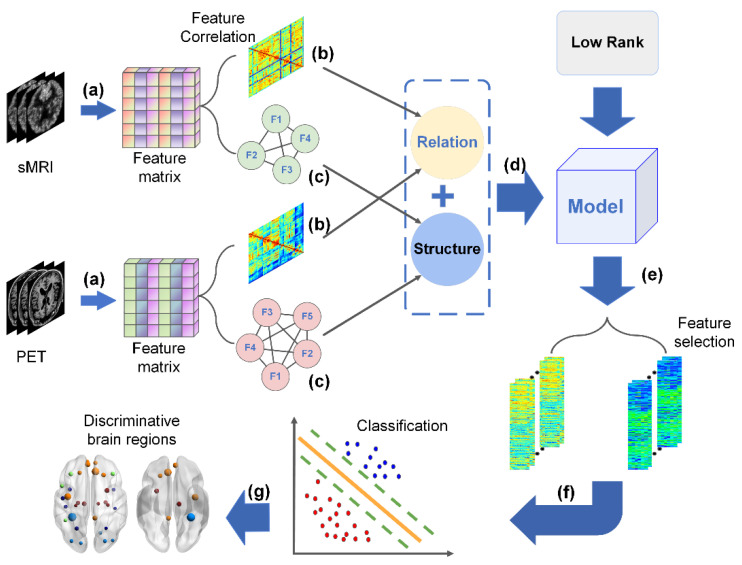
The research framework. (**a**) Original sMRI and PET images were preprocessed, then regions of interest were extracted by AAL template as sMRI and PET features, and the corresponding feature matrices of sMRI and PET were obtained, respectively. (**b**) Correlation coefficients between feature nodes of each modal data were calculated to obtain the feature correlation matrix, and the feature correlation regularization was obtained by linear fusion. (**c**) Feature matrix was weighted fusion, then the adjacent nodes were calculated to obtain the adjacency matrix, and the feature graph Laplacian matrix was constructed according to the cosine distance method to obtain the feature structure regularization. (**d**) The two regularizations were embedded into the multi-task model with low-rank constraint for feature selection. (**e**) Feature vectors with good characterization were selected by the proposed model, standardized respectively, and the features extracted from multi-modal data were linearly fused to obtain a new fused feature matrix. (**f**) The test set and training set were divided from the fused feature matrix, the training set was trained by the 10-fold cross-validation method and SVM to obtain the classification model, and the classification performance of the model was verified by the test set; (**g**) The corresponding discriminative brain regions of the selected feature nodes were visualized to analyze the discriminative brain regions affected by MCI and AD respectively.

Our research framework which is shown in Figure 1 mainly includes the following steps.

### 2.2. Data Acquisition and Preprocessing

The data were collected from Alzheimer’s Disease Neuroimaging Initiative (ADNI), which focuses on the prediction and diagnosis of AD. ADNI was approved by the Institutional Review Boards (IRBs), and all subjects were reviewed and approved by the IRBs within the ADNI study, meeting all ethical standards for data collection. Our study included structural magnetic resonance imaging (sMRI) and positron emission tomography (PET) imaging data of 73 normal controls (NCs), 53 EMCI subjects, 49 LMCI subjects, and 69 AD subjects. The specific information of subjects is shown in Table 1.

There are different inclusion and exclusion criteria for the four categories of subjects: For normal controls, cognition must be normal, and without memory impairment, the Mini-Mental State Exam (MMSE) score should be between 24 and 30, Clinical Dementia Rating (CDR) and Memory Box Score should be 0, while any normal controls with significant neurologic disease must be excluded. EMCI subjects must have subjective memory problems, MMSE score between 24 and 30, CDR and Memory Box score must both be 0.5; Subjects with any significant neurological disease other than suspected early Alzheimer’s disease need to be excluded. For LMCI subjects, the inclusion criteria were consistent with EMCI, and the criteria to distinguish EMCI and LMCI were determined by Wechsler Memory Scale. Exclusion criteria are consistent with EMCI. Patients with AD must have subjective memory problems, MMSE score between 20 and 26, and CDR must be 0.5 or 1. Probable AD needs to meet the NINCDS/ADRDA criteria [19]. Subjects with other neurological disorders besides Alzheimer’s had to be excluded.

SPM12 software [20] was used to preprocess sMRI and PET original images with voxel-based morphometric (VBM) analysis methods. For sMRI data, spatial standardization was first carried out. The MNI152 standard brain template was used to map the same region of each original image to the template region one by one, which helps to eliminate the brain differences caused by individual factors. Then, the image was segmented into gray matter, white matter, and cerebrospinal fluid, and the noise was eliminated by a smoothing operation. Finally, the AAL template [21] was used to extract the average gray matter density of the brain region of interest (ROI) as the sMRI data features. For PET data, the realignment of the images was carried out first. The images were coregistered onto the MNI152 brain space [21,22] for normalization and smoothing operation, and the width of 8 mm of a Gaussian filter was adopted. Finally, the glucose metabolism of the cerebral regions of interest was extracted using AAL template as PET data features.

### 2.3. Joint Feature Learning with Low-Rank Constraint

The multi-task learning model has been widely applied into multi-modal feature selection. Its main advantage is that it can mine deep common data features among different tasks and realize information sharing among multiple modal data [23]. $L_{2,1}$-norm regularizer can minimize the loss function while making weight vectors as sparse as possible, while selecting feature vectors with representation. Previous studies have shown that low-rank constraint can also find shared information well [24] and can measure the similarity between matrix row vectors. Therefore, low-rank constraint is introduced to capture the potential relationship between different task features. It promises to improve the information sharing between different tasks in the multi-task model and improve the model generalization performance. The following model is established:

(1) $$\underset{w_{1},w_{2},\ldots ,w_{m}}{\min}\sum_{i=1}^{m}{\left\|Y_{i}-X_{i}w_{i}\right\|}_{2}^{2}+\gamma rank(W)$$

where $X_{i}={\left[x_{1},x_{2},\ldots ,x_{N}\right]}^{T}\in {\mathbb{R}}^{N\times p}$ is the feature matrix of the *i*-th modality, N represents the number of subjects, p represents the number of features, which is also the number of regions of interest; $Y_{i}={\left[y_{1},y_{2},\ldots ,y_{N}\right]}^{T}\in {\mathbb{R}}^{N\times 1}$ represents the number of subjects labels in the *i*-th modality, $W=[w_{1},w_{2},\ldots ,w_{m}]\in {\mathbb{R}}^{p\times m}$ is the feature weight matrix, and each element in $w_{i}$ represents the corresponding feature weight value in the *i*-th modality, *m* is the number of the modality; rank(⋅) represents low-rank constraint, and *γ* is the low-rank constraint coefficient.

In fact, the low-rank constraint for a matrix is nonconvex and it is a typical NP-hard problem. It has been proved that trace norm can be used to approximate low-rank constraint [25,26]. Finally, the loss function of multi-modal feature learning based on low-rank constraint is obtained, as shown in Equation (2):

(2) $$\underset{w_{1},w_{2},\ldots ,w_{m}}{\min}\sum_{i=1}^{m}{\left\|Y_{i}-X_{i}w_{i}\right\|}_{2}^{2}+\gamma {\left\|W\right\|}_{*}$$

where ${\left\|\cdot \right\|}_{*}$ represents trace norm of the matrix, ${\left\|W\right\|}_{*}=\sum_{i}\lambda_{i}$ is the sum of all singular values of the matrix W.

### 2.4. Feature Correlation and Feature Structure Regularization

In multi-modal data, features are often related to each other [27]. Feature selection is to select a feature from several highly correlated features, when one feature is restricted, which will inevitably lead to the selection of highly correlated features [28]. Therefore, we consider the correlation of features between different modalities, the weighted average of the feature correlation matrix of various modalities. Finally, we propose a new feature correlation regularization, as shown in Equation (3):

(3) $$tr(W^{T}\sum_{i=1}^{m}R_{i}W)$$

where $R_{i}$ is the correlation coefficient matrix of the *i*-th modality, and tr(⋅) represents the trace of the matrix.

The common calculation methods of correlation coefficient include the Pearson correlation coefficient, the Spearman correlation coefficient, and the Kendall correlation coefficient. The Pearson correlation coefficient can measure the linear correlation of two variables and its value lies between −1 and 1. The Spearman correlation coefficient and Kendall correlation coefficient, compared with the Pearson correlation coefficient, have more relaxed requirements for data and wider application scope [29,30].

Furthermore, when the distance between two feature vectors is close in space, the distance between their corresponding weight vectors should also be close. Inspired by manifold learning and feature fusion [17,31,32,33], we use the weighted fusion multi-modal feature matrix to construct the Laplacian matrix to preserve the local geometric structure of features, so we have the following feature structure regularization:

(4) $$\begin{array}{l} \frac{1}{2}\sum_{j,k}^{p}h_{jk}{\left\|W_{j\cdot }-W_{k\cdot }\right\|}_{2}^{2}\\ =tr(W^{T}(S-H)W)\\ =tr(W^{T}L_{F}W) \end{array}$$

where $W_{j\cdot }$ and $W_{k\cdot }$ represent the *j*-th row and *k*-th row vectors of the weight matrix respectively, $H\in {\mathbb{R}}^{p\times p}$ represents an adjacency matrix of the features. $S\in {\mathbb{R}}^{p\times p}$ is a degree and diagonal matrix, the principal diagonal element is the degree of each feature node in the adjacency matrix H, and the calculation equation is $S_{ii}=\sum_{i=1}^{p}H_{i\cdot }$. $L_{F}=\in {\mathbb{R}}^{p\times p}$ represents the Laplacian matrix computed after the fusion of the feature matrices, and $L_{F}=S-H$.

For the adjacency matrix H, there are three construction methods: 0–1 weighting, heat-kernel function, and cosine distance [34]. We adopt the cosine distance method to construct, and its calculation equation is as follows:

(5) $$h_{ij}=\{\begin{array}{cc} \frac{X_{\cdot i}^{T}X_{\cdot j}}{\left\|X_{\cdot i}\right\|\left\|X_{\cdot j}\right\|}, & if\;X_{\cdot i}\;and\;X_{\cdot j}\;are\;adjacent\\ 0, & otherwise \end{array}$$

where $h_{ij}$ is the *i*-th row and *j*-th column element of the adjacency matrix H, which is used to measure the similarity between *i*-th and *j*-th columns of the feature vectors in the feature matrix X. $X_{\cdot i}$ and $X_{\cdot j}$ represent the feature vectors of the *i*-th and *j*-th columns of the feature matrix X, respectively.

### 2.5. Multi-Modal Feature Selection

In this work, an improved feature selection algorithm is proposed. Based on the multi-task learning model, trace norm is introduced to improve information sharing between different modalities, and the feature correlation regularization and feature structure regularization proposed above are introduced. The potential correlation between features is learned and the local geometric structure of features is preserved while minimizing the loss function, in order to improve the generalization ability of the model. Finally, we obtain the final established loss function:

(6) $$\underset{w_{1},w_{2},\ldots ,w_{m}}{\min}\sum_{i=1}^{m}{\left\|Y_{i}-X_{i}w_{i}\right\|}_{2}^{2}+\alpha tr(W^{T}\sum_{i=1}^{m}R_{i}W)+\beta tr(W^{T}L_{F}W)+\gamma {\left\|W\right\|}_{*}$$

where *α*, *β,* and *γ* are regularization parameters, and they are all real numbers greater than zero. The loss function is divided into four parts, the first is empirical error, the second is feature correlation regularization, the third is feature structure regularization, and the fourth is trace norm.

The solution of the objective loss function of Equation (6) is a convex optimization problem. Combining with the existing optimization algorithm [35,36], an optimization algorithm is proposed to solve this problem. First, the loss function is divided into a convex and non-convex function. The trace norm is a non-convex regularization term, and the remainder is convex terms. Let $\varphi (W)=\eta (W)+\gamma {\left\|W\right\|}_{*}$, where η(W) is the differentiable part, and the original loss function can be rewritten as:

(7) $$\underset{w_{1},w_{2},\ldots ,w_{m}}{\min}\eta (W)+\gamma {\left\|W\right\|}_{*}$$

For any given $W_{k-1}$, consider the second-order approximate form of φ(W) at $W_{k-1}$, and we obtain:

(8) $$\begin{array}{l} \varphi (W)\approx Q(W,W_{k-1})\\ =\eta (W_{k-1})+<W-W_{k-1},\nabla \eta (W_{k-1})>+\frac{s}{2}{\left\|W-W_{k-1}\right\|}_{F}^{2}+\gamma {\left\|W\right\|}_{*} \end{array}$$

where <⋅,⋅> denotes the inner product, ${\left\|\cdot \right\|}_{F}$ is the Frobenius norm of the matrix, $\nabla \eta (W_{k-1})$ represents the derivative of the differentiable function η(⋅) at $W_{k-1}$, and the iterative updating equation of the weight matrix W is further obtained:

(9) $$W_{k}=prox_{\gamma {\left\|\cdot \right\|}_{*}}(W_{k-1}-\frac{1}{s}\nabla \eta (W_{k-1}))$$

where *s* is the step length, and the calculation of the proximal operator $prox_{\gamma {\left\|\cdot \right\|}_{*}}(\cdot )$ is shown in Equation (10):

(10) $$prox_{\gamma {\left\|\cdot \right\|}_{*}}(Z)=\frac{1}{2}{\left\|W_{k}-Z\right\|}_{F}^{2}+\gamma {\left\|W\right\|}_{*}$$

For the solution of Equation (10), according to the conclusions of existing studies [37,38], it can be computed by singular value decomposition (SVD) of $W_{k-1}-\frac{1}{s}\nabla \eta (W_{k-1})$, as shown in Equation (11):

(11) $$prox_{\gamma {\left\|\cdot \right\|}_{*}}(Z)=UD_{\gamma }V^{T}$$

where $UD_{\gamma }V^{T}$ is the SVD of Z, $D_{\gamma }$ is a diagonal matrix, and the diagonal element of $D_{\gamma }$ is ${\left(D_{\gamma }\right)}_{ii}=\max\{D_{ii}-\gamma ,0\}$. Equation (11) can make the weight matrix W become low-rank while shrinking the singular values.

In the above optimization algorithm, the time complexity of this algorithm can still be achieved O(1/M), where M is the maximum number of iterations of the algorithm, despite the existence of a non-differentiable trace norm approximate low-rank constraint. Meanwhile, we summarize the optimization algorithm flow of loss function to show the above iterative update process more clearly, as shown in Table 2.

### 2.6. Classification and Evaluation Measures

SVM is suitable for binary classification with small subjects. It has good generalization ability, and can avoid dimensional disasters, and is often applied to disease classification [39,40,41]. In our study, the loss function of Equation (6) is used for feature selection, and the multi-modal features obtained are linearly fused. Then the features are input into the SVM to classify MCI and AD, and the performance of the model is estimated from different classification indexes.

This work mainly includes six indicators to evaluate the classification performance. The first four common classification indicators are accuracy (ACC), area under curve (AUC), sensitivity (SEN), specificity (SPE). Meanwhile, the geometric mean (GMean) and F1 Score (F1) are used to further measure classification performance to overcome the influence of different proportions of positive and negative subjects on the classification results.

Each indicator is defined as follows:

(12) $$\mathrm{ACC}=\frac{\mathrm{TN}+\mathrm{TP}}{\mathrm{TP}+\mathrm{FP}+\mathrm{TN}+\mathrm{FN}}$$

(13) $$\mathrm{AUC}=P(P_{-}<P_{+})$$

(14) $$\mathrm{SEN}=\frac{\mathrm{TP}}{\mathrm{TP}+\mathrm{FN}}$$

(15) $$\mathrm{SPE}=\frac{\mathrm{TN}}{\mathrm{TN}+\mathrm{FP}}$$

(16) $$\mathrm{GMean}=\sqrt{\frac{\mathrm{TP}}{\mathrm{TP}+\mathrm{FN}}+\frac{\mathrm{TN}}{\mathrm{TN}+\mathrm{FP}}}$$

(17) $$F1=\frac{2\mathrm{TP}}{2\mathrm{TP}+\mathrm{FP}+\mathrm{FN}}$$

where TP, TN, FP, and FN represent true positive, true negative, false positive, and false negative respectively. Accuracy (ACC) represents the proportion of correctly classified subjects to all subjects, sensitivity (SEN) and specificity (SPE) describe the proportion of positive and negative subjects that are correctly classified, respectively, while area under the curve (AUC) describes the area of the ROC curve.

## 3. Results

### 3.1. Classification Performance

In the experiment, four methods were selected for comparison and respectively applied to MCI and AD classification. The classification performance of each method is shown in Table 3. The baseline method is the widely used Lasso feature selection method [12], CMTL [42] is a multi-task learning method based on clustering, MTFS [2] is a multi-task feature selection method with $L_{2,1}$-norm regularizer and applied to AD, and HMTFS [18] is a multi-modal feature selection method based on MTFS that introduces hypergraph. In addition to being less sensitive in NC vs. LMCI classification, the proposed method has a better classification performance for NC vs. AD, NC vs. EMCI and EMCI vs. LMCI than previous methods. Compared to the four comparison methods, feature correlation regularization can better reflect the potential relationship between multiple features, while feature structure regularization can preserve the local geometric structure of features.

In NC vs. AD classification, ACC, AUC, SEN, SPE, GMean and F1 reach 91.85 ± 1.42%, 92.84 ± 1.69%, 91.07 ± 2.02%, 92.27 ± 2.12%, 91.23 ± 1.77%, and 91.81 ± 1.59%, respectively. Compared with the other four methods, the classification performance is improved. It is worth noting that the method improved greatly in NC vs. EMCI, and the six classification indexes achieve 78.29 ± 2.20%, 78.03 ± 2.41%, 82.02 ± 2.13%, 74.73 ± 3.04%, 77.18 ± 2.58%, and 81.00 ± 1.93%, respectively. MTFS has a better classification performance than Lasso and CMTL, indicating that the introduction of $L_{2,1}$-norm regularizer can effectively sparse multi-modal features and capture effective features, which is consistent with the research results of Zhang et al. [2]. In addition, HMTFS has better classification performance than MTFS, indicating that introducing hypergraph regularization can indeed discover high-order relations between subjects, which is consistent with the research results of Shao et al. [18]. Besides, FC2FS has better classification performance than HMTFS, indicating that feature correlation and feature structure regularization can effectively discover more potential features and improve classification performance, which proves the effectiveness of the method.

**Figure 2:**
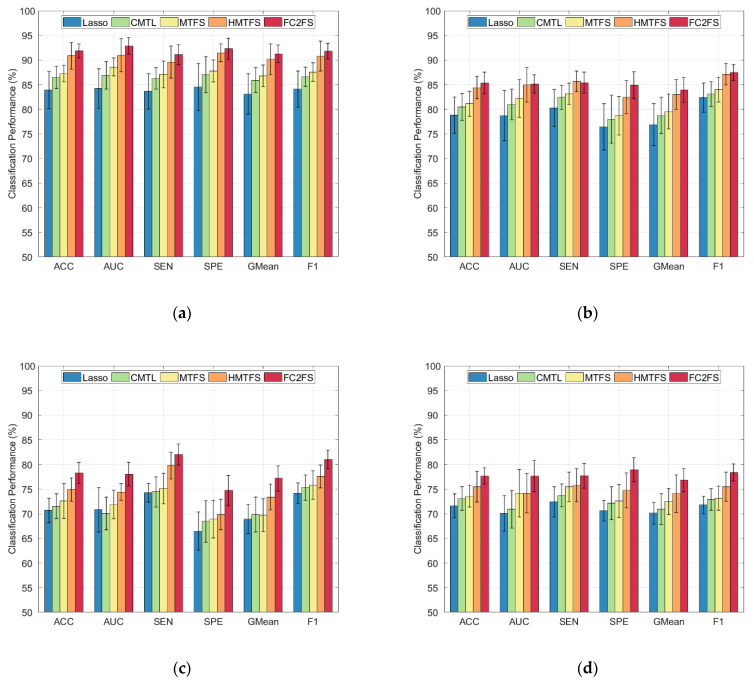
Classification performance of different methods. (**a**) NC vs. AD, (**b**) NC vs. LMCI, (**c**) NC vs. EMCI, (**d**) EMCI vs. LMCI.

We used bar charts to represent the classification performance of the five methods more vividly on MCI and AD, aiming at better display of the experiment results of different methods, as shown in Figure 2.

### 3.2. Parameter Sensitivity and Correlation Analysis

The selection of different parameters has different effects on the experiment results, and the parameter selection directly affects the performance of the method for MCI and AD classification. This section mainly analyzes the various influences of the main parameters involved in the experiment and calculates the correlation coefficient on the experimental results, while exploring the optimal parameter selection of the experiment. The influence of the weighted fusion coefficient of the feature matrix, two kinds of regularization parameters, and the common calculation methods of the feature correlation coefficient on the classification performance are analyzed. Ten-fold cross-validation was adopted to make the experiment results credible [43]. According to the results of each experiment, the mean value of the ten experiment results was calculated randomly as the value of the classification performance index.

#### 3.2.1. The Influence of Fusion Coefficient on Classification Accuracy

**Figure 3:**
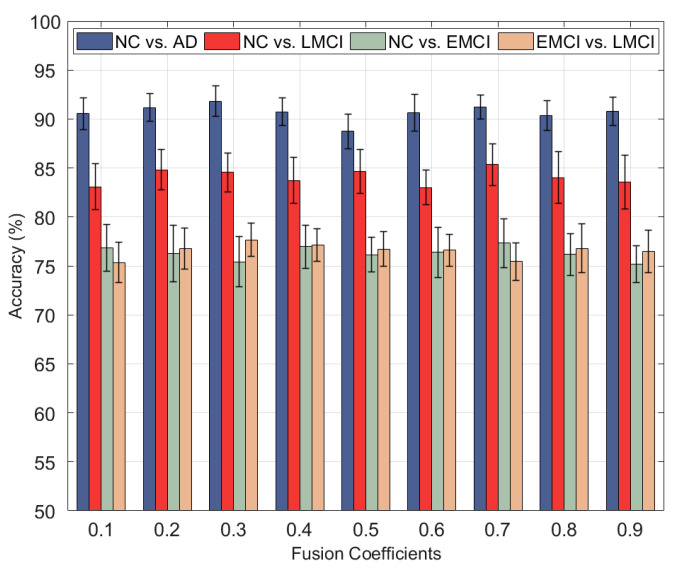
Influence of different fusion coefficients on classification accuracy.

First, *α*, *β,* and *γ* values were fixed, *α* and *β* were set to $2^{-2}$ and *γ* to $2^{-1}$, and the Pearson correlation was used to calculate the correlation coefficient matrix. Then the feature matrix fusion coefficient τ of sMRI data was set, with the range of change of 0 to 1 and step size of 0.1 decimal, then giving the feature matrix fusion coefficient of PET data as 1−τ. Further, the influence of fusion with different fusion coefficients on classification performance was explored, and experimental results were obtained as shown in Figure 3. Among them, the optimal fusion coefficient of NC vs. AD and EMCI vs. LMCI is 0.3, while the optimal fusion coefficient of NC vs. LMCI and NC vs. EMCI is 0.7. Using different fusion coefficient combinations to fuse the feature matrix directly affects the classification accuracy. In addition, the analysis of the four experiment results shows that in NC vs. AD and EMCI vs. LMCI classification, the sMRI fusion coefficient is 0.3, while the PET fusion coefficient is 0.7, indicating that sMRI data contributes more to classification performance than PET data. In NC vs. LMCI and NC vs. EMCI, the sMRI fusion coefficient was 0.7, while the PET fusion coefficient was 0.3, indicating that sMRI data had more influence on classification performance than PET data.

#### 3.2.2. Effects of Regularization Parameters on Classification Performance

**Figure 4:**
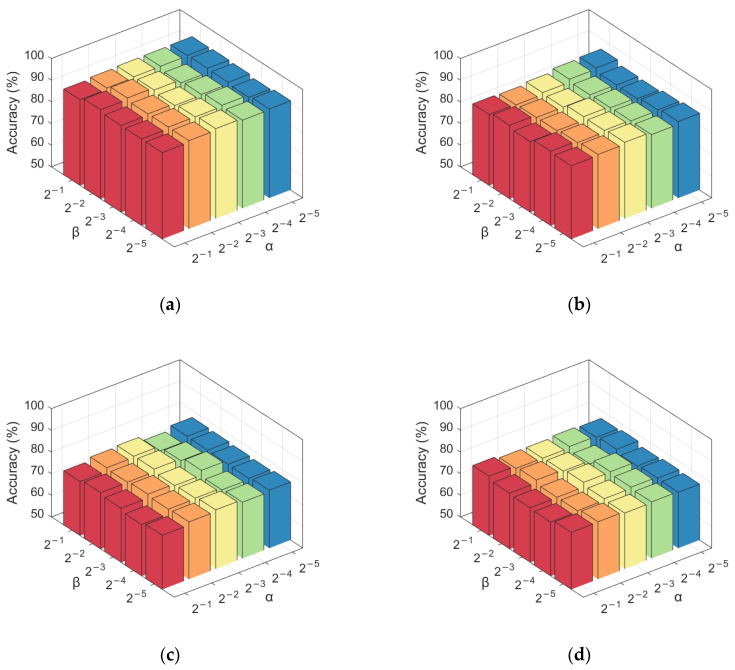
Influence of regularization parameters on classification accuracy. (**a**) NC vs. AD, (**b**) NC vs. LMCI, (**c**) NC vs. EMCI, (**d**) EMCI vs. LMCI.

In the established feature selection model, there are three regularization parameters, namely *α*, *β,* and *γ*. In the experiment, the appropriate *γ* value was first selected, and the value range of *α* and *β* was set as $\left\{2^{-1},2^{-2},2^{-3},2^{-4},2^{-5}\right\}$ to explore the influence of different regularization parameter combinations on classification accuracy and reduce the time complexity of the model. Then the values of each group of *α* and *β* were fixed. Finally, the classification accuracy of each group of values was calculated using the 10-fold cross-validation method, and the results were obtained as shown in Figure 4. Through analysis, it can be found that the classification accuracy does not fluctuate greatly under different regularization parameter combinations, which indicates that the method has a certain stability. In most cases, for each fixed *α* (*β*), as the value of *β* (*α*) decreases, the classification accuracy generally shows a trend of increasing first and then decreasing. The reason may be that with the decrease of the regularization parameter value, the weight of the feature correlation regularization and feature structure regularization decreases. The feature selection model’s ability to capture the correlation between features is weakened, which results in partial effective features being ignored and reduced accuracy.

#### 3.2.3. The Influence of Correlation Coefficient Calculation Methods on Classification Performance

**Figure 5:**
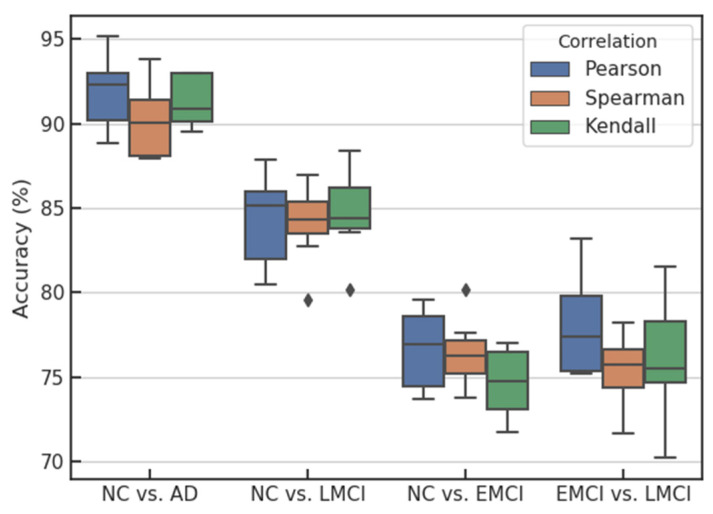
Influence of correlation coefficient calculation on classification accuracy.

The influence of the proposed method on classification accuracy was analyzed when different methods were applied to calculate the feature correlation coefficients. The Pearson correlation coefficient, Spearman correlation coefficient and Kendall correlation coefficient were used to calculate the influence of correlation between features on classification accuracy under the three conditions, as shown in Figure 5. The results show that when the Pearson correlation coefficient is used to average the feature correlation matrix, the median accuracy obtained is always higher than that calculated by using the other two correlation coefficients, and the Pearson correlation has a larger fluctuation range than the other two correlation coefficients. The main reason may be that the Pearson correlation coefficient is sensitive to outliers. When more outliers of the feature correlation coefficient are generated, the Pearson correlation coefficient is greatly affected, while the Spearman correlation coefficient and the Kendall correlation coefficient are correlation coefficients based on matrix rank, so they are robust to outliers.

### 3.3. Discriminative Brain Regions

**Figure 6:**
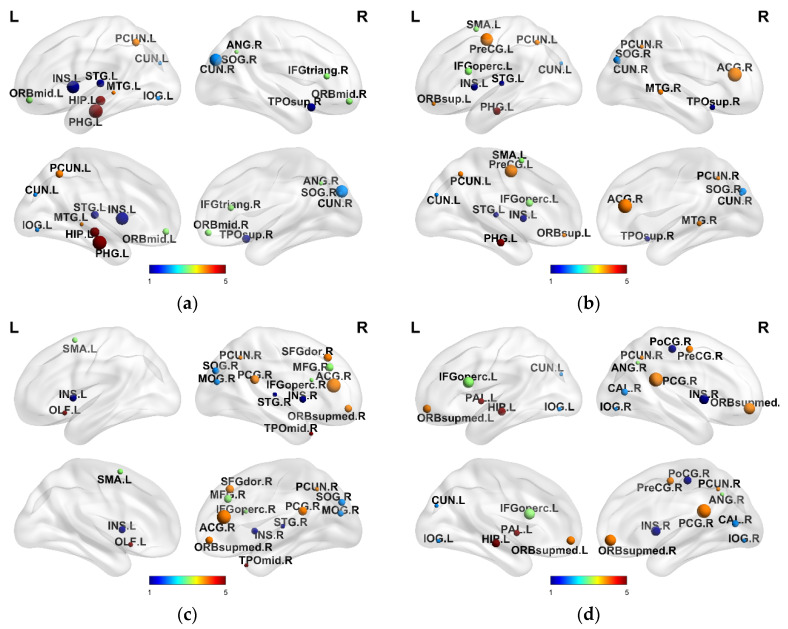
Visualization of discriminative brain regions. (**a**) NC vs. AD, (**b**) NC vs. LMCI, (**c**) NC vs. EMCI, (**d**) EMCI vs. LMCI.

The optimal regularized parameters determined by the 10-fold cross-validation method were selected in the experiment to find the most discriminant biologic features in MCI and AD classification. Further statistics obtain the brain regions corresponding to the top 15 feature vectors with different classification results. These brain areas are called discriminative brain regions, as shown in Table 4. The influence degree of MCI and AD on brain regions was discussed, and the BrainNet Viewer toolbox [44] was used to visually display the selected discriminative brain regions, as shown in Figure 6. As can be seen from the obtained results, most of the selected discriminative brain regions in NC vs. AD and NC vs. LMCI classification were confirmed, while only a small part of the selected discriminative brain regions in NC vs. EMCI and EMCI vs. LMCI classification was confirmed by previous studies. This phenomenon explains the lower performance of the latter classification.

By analyzing the brain regions obtained by NC vs. AD and NC vs. LMCI classification, we can find that among the selected discriminative brain regions, the ones belonging to temporal lobe, prefrontal lobe and occipital lobe account for a large proportion of the first 15 discriminative brain regions. NC vs. EMCI and EMCI vs. LMCI classification results showed that the discriminative brain regions belonging to the prefrontal lobe and occipital lobe accounted for a large proportion. The temporal lobe region mainly includes five discriminative brain regions: the left superior temporal gyrus (STG.L), the left middle temporal gyrus (MTG.L), the left hippocampus (HIP.L), the left parahippocampal gyrus (PHG.L), and the right temporal pole superior temporal gyrus (TPOsup.r). The temporal lobe is closely related to language and memory, among which damage to the left superior temporal gyrus (STG.L) will cause sensory aphasia, while damage to the left hippocampus (HIP.L) and left parahippocampus (PHG.L), one of the important organs in the brain involved in learning and memory storage, will lead to atrophy and memory impairment. Relevant studies have confirmed that the volume and morphology of the AD hippocampus will change compared with normal subjects [61,62]. The prefrontal lobe has the function of managing cognition, emotion, and behavior, which is mainly related to motor and higher mental function, while occipital lobe lesions will not only lead to visual impairment but are also accompanied by memory and motor defects. The selected discriminative brain regions belonging to the prefrontal lobe and occipital lobe, mainly include the right middle frontal gyrus (MFG.R), left middle frontal gyrus, orbital part (ORBmid.L), left superior orbital cortex (ORBsup.L), right inferior frontal gyrus, triangular part (IFGtriang.R), left opercular part of inferior frontal gyrus (IFGoperc.L), right anterior cingulate gyrus (ACG.R), left insula (INS.L), left cuneus (CUN.L), and right cuneus (CUN.R).

Notably, the right posterior cingulate gyrus (PCG.r) and the left precuneus (PCUN.L) were selected in NC vs. EMCI classification. These two discriminative brain regions are associated with the process of memory formation, indicating that compared with normal subjects, memory has been changed during the EMCI stage. The right angular gyrus (ANG.R) was identified in the EMCI vs. LMCI classification and is an important biological feature that distinguishes the first two [54]. However, there are still a small number of brain regions that have not been confirmed by previous studies among the selected discriminative brain regions. This may be caused by the fact that some of these brain regions do indeed have a strong impact on MCI and AD classification, but the existing relevant studies have not proved it. In addition, there may still be a few redundant features in the feature selection, leading to the selection of brain regions weakly related to the disease.

## 4. Discussion

There are few studies on the relationship and structure between feature nodes among the existing multi-modal feature selection methods for diseases. Most of the methods focusing on the relationship between the same modality or different modality subjects, do not consider the influence of the relationship between feature nodes and structure on the model, and lack interpretation. For example, Jie et al. [17] used manifold learning to measure the distance between different subjects to maintain the adjacent structure between subjects, and applied it to MCI classification, achieving a good classification performance and verifying the effectiveness of the method. However, this method ignores the similarity relation between feature nodes and local geometry structure and lacks explanation for feature selection. 

The results of this experiment show that this feature relation cannot be ignored in MCI and AD classification and has a positive influence on feature selection. It is worth mentioning that in the study of Lei et al. [63], four feature relations were regularized and the $L_{2,1}$-norm regularizer was introduced to sparse feature weight vectors, and it was finally applied to the classification of Parkinson’s disease, achieving good classification performance and good interpretability. Yet, this method has some disadvantages, the obvious one is that there are too many model parameters, and the time complexity of the method increases in practical application. In our study, the multi-modal feature selection method with feature correlation and feature structure fusion fully considers the internal connection between feature nodes, solves the problem of too many parameters in the loss function, reduces the time complexity of feature selection, and brings better classification performance.

Additionally, it is worth noting that we extract feature vectors directly from the original sMRI and PET images to obtain the feature matrix, and each feature vector represents a different brain region in the AAL template. When learning feature weight, the loss function essentially learns the weight value of the brain region through the training set and selects the corresponding feature vectors of the brain regions that are helpful to improve the classification performance. Therefore, the proposed method improves the interpretability of the model.

In summary, our work has potential clinical application value in clinical diagnosis. On the one hand, since the scale is subjective in clinical use [64] and the determination of patients with cognitive impairment is also personal, applying this method in the clinic will reduce human intervention, assist clinical diagnosis, and make diagnostic results more objective. On the other hand, the experimental results showed that the sensitivity and specificity of the method were significantly improved in the classification of NC and EMCI, which is clinically significant [65], reducing the risk of misdiagnosis of normal controls as early cognitive impairment patients require timely drug intervention. At the same time, the experiment proved that the method is more suitable for accurately capturing and identifying patients with subtle changes in brain regions. This property is better suited for diagnosing more difficult cognitive impairment associated with certain diseases, such as End-Stage Renal Disease (ESRD) combined with cognitive impairment [66], the exact neuropathological mechanism of which is still unclear. Cognitive impairment is a comorbidity of ESRD, and treatment of ESRD may also change brain function and structure [67], making it more challenging to identify MCI. In the future, based on the proposed model, we will further explore the identification of ESRD patients with cognitive impairment.

## 5. Conclusions

In this study, a multi-modal feature selection algorithm with feature correlation and feature structure fusion is proposed and applied to MCI and AD classification. In this method, low-rank constraint is introduced based on multi-task learning, moreover, feature correlation and feature structure regularization are adopted considering feature node relations. Finally, feature learning is carried out according to the constructed loss function. Experimental results showed that the proposed method performed better than the comparison methods in classification performance.

Nevertheless, our work has some limitations. When constructing the feature correlation coefficient matrix, only the relatively common calculation method of the correlation coefficient was considered, and the method that can better measure the correlation between two features or even multiple feature nodes remains to be discussed. Moreover, only the linear fusion of multi-modal features was input into the SVM classifier during classification. In the future, the integration model [68] deserves to be discussed to combine several weak classifiers into a strong classifier, and the classification performances of MCI and AD need to be further improved.

## Figures and Tables

**Table 1:** The subject’s information.

Characteristic	Normal	EMCI	LMCI	AD
Number	73	53	49	69
Male/Female	39/34	23/30	22/27	36/33
Age (mean ± SD)	75.9 ± 6.79	72.9 ± 7.6	73.1 ± 8.23	72.54 ± 6.12
MMSE (mean ± SD)	28.14 ± 1.21	27.1 ± 1.62	26.3 ± 2.1	22.54 ± 2.16

**Table 2:** Optimization algorithm of loss function.

Line No.	Optimization Algorithm of Loss Function
1	Input: $X_{i}\in {\mathbb{R}}^{N\times p}$ represents the feature matrix of the *i*-th modality; $Y_{i}\in {\mathbb{R}}^{N\times 1}$ represents the label corresponding to the *i*-th modality subjects.
2	Output: $W\in {\mathbb{R}}^{p\times m}$ represents the weight matrix of the feature.
3	Normalize feature matrix $X_{i}$ and initialize $W_{0}$, s;
4	Compute feature correlation matrix of *i*-th modality, and weighted average;
5	Weighted fusion of the feature matrix, and compute feature Laplacian matrix $L_{F}$;
6	Do
7	Compute SVD of $W_{k-1}-\frac{1}{s}\nabla \eta (W_{k-1})$;
8	Update $Z_{k-1}=W_{k-1}-\frac{1}{s}\nabla \eta (W_{k-1})$;
9	Update $W_{k}=prox_{\gamma {\left\|\cdot \right\|}_{*}}(Z_{k-1})$;
10	Update step length s;
11	While it reaches the maximum number of iterations or converges

**Table 3:** Classification performance of different methods.

Task	Methods	ACC (%) ±STD	AUC (%) ±STD	SEN (%) ±STD	SPE (%) ±STD	GMean (%) ±STD	F1 (%) ±STD
NC vs. AD	Lasso	83.89 ± 3.80	84.18 ± 4.00	83.60 ± 3.55	84.50 ± 4.76	83.09 ± 4.06	84.08 ± 3.67
	CMTL	86.44 ± 2.26	86.86 ± 2.79	86.24 ± 2.14	86.99 ± 3.62	85.91 ± 2.51	86.59 ± 1.98
	MTFS	87.28 ± 1.66	88.58 ± 1.87	87.09 ± 2.70	87.76 ± 2.26	86.75 ± 2.21	87.52 ± 1.88
	HMTFS	90.83 ± 2.72	90.96 ± 3.38	89.54 ± 3.24	91.45 ± 1.80	90.14 ± 3.12	90.78 ± 3.01
	FC2FS	91.85 ± 1.42	92.84 ± 1.69	91.07 ± 2.02	92.27 ± 2.12	91.23 ± 1.77	91.81 ± 1.59
NC vs. LMCI	Lasso	78.81 ± 3.69	78.66 ± 5.09	80.24 ± 3.75	76.41 ± 4.71	76.85 ± 4.28	82.36 ± 2.97
	CMTL	80.45 ± 2.72	80.96 ± 3.09	82.38 ± 2.40	77.96 ± 4.87	78.75 ± 3.68	83.09 ± 2.53
	MTFS	81.10 ± 2.55	82.20 ± 3.84	83.14 ± 2.18	78.65 ± 3.89	79.52 ± 3.51	83.96 ± 2.49
	HMTFS	84.39 ± 2.30	84.96 ± 3.52	85.65 ± 2.07	82.42 ± 3.36	83.00 ± 3.02	87.09 ± 2.17
	FC2FS	85.33 ± 2.22	85.11 ± 1.86	85.38 ± 2.13	84.85 ± 2.73	83.91 ± 2.55	87.45 ± 1.59
NC vs. EMCI	Lasso	70.67 ± 2.47	70.81 ± 4.54	74.24 ± 1.91	66.47 ± 3.89	68.90 ± 2.96	74.18 ± 2.10
	CMTL	71.52 ± 2.48	70.06 ± 3.34	74.44 ± 3.07	68.44 ± 4.21	69.82 ± 3.56	75.30 ± 2.56
	MTFS	72.56 ± 3.55	71.86 ± 2.86	75.10 ± 3.11	68.89 ± 3.83	69.69 ± 3.30	75.80 ± 2.89
	HMTFS	74.90 ± 2.41	74.41 ± 1.68	79.77 ± 2.72	69.86 ± 3.11	73.37 ± 2.61	77.56 ± 2.32
	FC2FS	78.29 ± 2.20	78.03 ± 2.41	82.02 ± 2.13	74.73 ± 3.04	77.18 ± 2.58	81.00 ± 1.93
EMCI vs. LMCI	Lasso	71.58 ± 2.43	70.07 ± 3.62	72.41 ± 3.06	70.65 ± 2.11	70.09 ± 2.20	71.77 ± 1.76
	CMTL	73.08 ± 2.37	70.89 ± 3.81	73.71 ± 2.31	72.15 ± 3.32	70.95 ± 3.12	72.91 ± 2.23
	MTFS	73.54 ± 2.22	74.14 ± 4.84	75.45 ± 2.98	72.58 ± 3.29	72.48 ± 2.62	73.16 ± 2.49
	HMTFS	75.46 ± 3.12	74.18 ± 3.97	75.77 ± 3.36	74.74 ± 3.53	74.08 ± 3.78	75.46 ± 2.98
	FC2FS	77.67 ± 1.65	77.63 ± 3.17	77.72 ± 2.54	78.94 ± 2.47	76.83 ± 2.35	78.35 ± 1.75

**Table 4:** Discriminative brain regions.

**NC vs. AD**				**NC vs. LMCI**			
**ID**	**Regions**	**Abbreviation**	**References**	**ID**	**Regions**	**Abbreviation**	**References**
29	Insula_L	INS.L	Jon et al. [45] Bi et al. [46]	11	Frontal_Inf_Oper_L	IFGoperc.L	Liu et al. [47]
81	Temporal_Sup_L	STG.L	Liu et al. [48]	39	ParaHippocampal_L	PHG.L	Lee et al. [49]
45	Cuneus_L	CUN.L	Le et al. [50]	29	Insula_R	INS.R	Li et al. [51]
10	Frontal_Mid_Orb_R	ORBmid.R		32	Cingulum_Ant_R	ACG.R	Li et al. [51]
67	Precuneus_L	PCUN.L	Bailly et al. [52]	1	Precentral_L	PreCG.L	Cai et al. [53]
85	Temporal_Mid_L	MTG.L	Liu et al. [48]	5	Frontal_Sup_Orb_L	ORBsup.L	Li et al. [51]
37	Hippocampus_L	HIP.L	Salvatore et al. [54]	19	Supp_Motor_Area_L	SMA.L	
53	Occipital_Inf_L	IOG.L		45	Cuneus_L	CUN.L	Le et al. [50]
50	Occipital_Sup_R	SOG.R		46	Cuneus_R	CUN.R	Le et al. [50]
39	ParaHippocampal_L	PHG.L	Katsel et al. [55]	50	Occipital_Sup_R	SOG.R	
84	Temporal_Pole_Sup_R	TPOsup.R	Salvatore et al. [54]	67	Precuneus_L	PCUN.L	Bailly et al. [52]
66	Angular_R	ANG.R		68	Precuneus_R	PCUN.R	Bailly et al. [52]
46	Cuneus_R	CUN.R	Le et al. [50]	81	Temporal_Sup_L	STG.L	
14	Frontal_Inf_Tri_R	IFGtriang.R	Salvatore et al. [54]	84	Temporal_Pole_Sup_R	TPOsup.R	Salvatore et al. [54]
9	Frontal_Mid_Orb_L	ORBmid.L	Zhang et al. [56]	86	Temporal_Mid_R	MTG.R	
**NC vs. EMCI**				**EMCI vs. LMCI**			
**ID**	**Regions**	**Abbreviation**	**References**	**ID**	**Regions**	**Abbreviation**	**References**
88	Temporal_Pole_Mid_R	TPOmid.R		68	Precuneus_R	PCUN.R	Lee et al. [49]
12	Frontal_Inf_Oper_R	IFGoperc.R	Chen et al. [57]	75	Pallidum_L	PAL.L	
82	Temporal_Sup_R	STG.R	Lee et al. [49]	37	Hippocampus_L	HIP.L	Wu et al. [58]
29	Insula_L	INS.L	Anna et al. [59]	66	Angular_R	ANG.R	Lee et al. [49]
32	Cingulum_Ant_R	ACG.R		2	Precentral_R	PreCG.R	
50	Occipital_Sup_R	SOG.R		11	Frontal_Inf_Oper_L	IFGoperc.L	
68	Precuneus_R	PCUN.R	Lee et al. [49]	25	Frontal_Mid_Orb_L	ORBsupmed.L	
4	Frontal_Sup_R	SFGdor.R		30	Insula_R	INS.R	Bi et al. [46]
8	Frontal_Mid_R	MFG.R	Lee et al. [49]	36	Cingulum_Post_R	PCG.R	
19	Supp_Motor_Area_L	SMA.L		44	Calcarine_R	CAL.R	
21	Olfactory_L	OLF.L	Vasavada et al. [60]	53	Occipital_Inf_L	IOG.L	
26	Frontal_Mid_Orb_R	ORBsupmed.R		54	Occipital_Inf_R	IOG.R	
30	Insula_R	INS.R	Anna et al. [59]	26	Frontal_Mid_Orb_R	ORBsupmed.R	
36	Cingulum_Post_R	PCG.R		45	Cuneus_L	CUN.L	
52	Occipital_Mid_R	MOG.R		58	Postcentral_R	PoCG.R	
